# Exploring the Relationship of Health Beliefs and Self-Care Behaviors Related to Diabetic Foot Ulcers of Type II Diabetes Mellitus Patients: A Cross-Sectional Study

**DOI:** 10.3390/ijerph18137207

**Published:** 2021-07-05

**Authors:** Meng-Chien Tsai, Hsiao-Ling Chuang, Cheng-Yi Huang, Shu-Hsin Lee, Wen-Chun Liao, Meng-Chih Lee, Ching-Pyng Kuo

**Affiliations:** 1 Department of Nursing, Ministry of Health and Welfare Taichung Hospital, Taichung 40343, Taiwan; Meng0701jian@gmail.com; 2 School of Nursing, Chung Shan Medical University, Taichung 40201, Taiwan; ling0911@csmu.edu.tw (H.-L.C.); huangcy@csmu.edu.tw (C.-Y.H.); shl@csmu.edu.tw (S.-H.L.); 3 Department of Nursing, Chung Shan Medical University Hospital, Taichung 40201, Taiwan; 4 School of Nursing, China Medical University, Taichung 406040, Taiwan; wcl@mail.cmu.edu.tw; 5 Department of Nursing, China Medical University Hospital, Taichung 40447, Taiwan; 6 Department of Nursing, Asia University, Taichung 41354, Taiwan; 7 Department of Family Medicine, Taichung Hospital, Ministry of Health and Welfare, Taichung 40343, Taiwan; 8 Institute of Medicine, Chung Shan Medical University, Taichung 40201, Taiwan; 9 Institute of Population Health Sciences, National Health Research Institutes, Miaoli 35053, Taiwan; 10 College of Management, Chaoyang University of Technology, Taichung 413310, Taiwan

**Keywords:** diabetes, health behavior, health belief, self-care, foot ulcer

## Abstract

Objective: Diabetic foot ulcers are an invasive complication of diabetes and are increasing. This study investigates the relationship between health beliefs and foot self-care behaviors, among people with type II diabetes. Methods: A cross-sectional design was adopted, and 98 patients were recruited from outpatient clinics of the endocrine department. The questionnaires of Demographic, Diabetes Foot Ulcer Health Belief Scale (Health Beliefs, DFUHBS), and Diabetes Foot Self-Care Behavior Scale (Self Care, DFSBS) were used to collect data. Results: Among the subjects living alone or who had diabetes less than ten years, the score of DFSBS was significantly lower than among those living with families or who had diabetes for ten years or more. The frequency of performing diabetes foot self-care behavior, among males was lower than among females significantly. Although there was no significant difference in the Health Belief total score, there were differences in the benefit subscale. Those who had junior high school level or less or had diabetes less than ten years, their score was significantly lower than those with senior high school level or more or had diabetes ten years or more. In a multivariable regression model, living with family, diabetes duration, and health beliefs explained 42.9% of the variance of diabetic-foot self-care behaviors. Conclusions: Living alone, shorter duration of diabetes, male gender, and lower health belief scores predict less adequate diabetic foot self-care behavior. Health care providers should assess these factors when designing individual care plans.

## 1. Introduction

One of the most common complications of diabetes mellitus is diabetic foot ulcers (DFU). According to the CDC, in 2016 among persons with diabetes aged 18 years or older in the U.S., the incidence of hospitalization for lower-extremity amputation was 5.6 per 1000 [1]. During the period from 2010 to 2015 in the U.S., diabetes mellitus accounted for an eleven-fold increase in hospital admissions and a ten-fold increase in hospital costs associated with foot ulcers [2].

Diabetic patients have a 15–25% frequency of developing foot ulcers during their lifetime. The five-year mortality rate of diabetic patients with foot ulcers is 2.5 times that of patients without foot ulcers [3]. In Taiwan, the probability of diabetic patients developing foot necrosis and infection is nearly 17 times higher than among people without diabetes [4]. According to the International Diabetes Federation’s 2015 Diabetes Atlas, it was estimated that there were 40–60 million patients with diabetic foot ulcers worldwide [5].

The clinical expenses related to diabetic foot ulcers and their complications are more significant than many cancers [3,6]. Studies show that poor control of blood glucose, peripheral neuropathy, and peripheral angiopathy can predict diabetic foot ulcers incidence. However, self-care behavior for the diabetic foot can prevent foot ulcers or the deterioration that they cause [7,8]. Diabetic patients’ self-management and self-care behavior skills can control the severity and complications of diabetes [9]. Researchers have explored the assessment tool of diabetic foot self-care behavior evaluation contents and cross-cultural considerations in recent years [9,10]. However, several studies have indicated that some diabetic patients’ self-care behavior for their feet is insufficient, they never examine their feet or never dry their toes after washing [7,11].

Diabetic patients’ health beliefs significantly influence their health behavior and effectively predict their self-care behavior [12,13,14]. However, few studies have specifically explored the relationship between diabetic foot ulcer patients’ health beliefs and foot self-care. Therefore, this study investigates the relationship between health beliefs related to diabetic foot ulcers and foot self-care behaviors of type II diabetic patients in Taiwan.

## 2. Methods

### 2.1. Research Design and Subjects

This study adopted a cross-sectional design to investigate the relationship between foot self-care behavior, demographics, disease characteristics, and health beliefs related to diabetic foot ulcers among Type II diabetic patients.

The sample inclusion criteria were the following: (1) patients diagnosed with type II diabetes; (2) age greater than 20 years old; (3) clear consciousness and the ability to communicate in both Mandarin Chinese and Taiwanese and; (4) agreement to participate in the study. In addition, patients diagnosed with type I diabetes, exocrine pancreatic disease, or severe cardiovascular disease, and those who had severe depression or anxiety, cognitive impairment were excluded from this study. Finally, we recruited a total of 98 patients from outpatient clinics of the endocrine department.

### 2.2. Measures

#### 2.2.1. Demographics and Disease Attributes

The demographic variables included age, gender, education level, occupation, residence (living with family or living alone), and marital status. Disease attributes included diabetic duration, body mass index (BMI), and glycated hemoglobin (HbA1c).

#### 2.2.2. Diabetic Foot Ulcer Health Beliefs Scale (DFUHBS)

This study adopted the DFUHBS developed by Chin et al. (2012) for data collection. This scale was based on Becker’s health belief model as a framework [15]. The scoring was based on a five-point Likert scale. There were three dimensions: threat beliefs (12 questions), benefit beliefs (9 questions), and barrier beliefs (14 questions). A higher score means a stronger health belief. A study of the DFUHBS in Taiwanese and had been validated. The Cronbach’s alpha values of the internal consistency were 0.88 in the total scale and 0.80 to 0.88 in the three subscales [16]. Internal consistency of the present study, from 0.88 to 0.92, was acceptable for both the three subscales and the total scale. 

#### 2.2.3. Diabetes Foot Self-Care Behavior Scale (DFSBS)

This study used the DFSBS developed by Chin and Huang (2013). There were seven questions on this scale, divided into two subscales: Execution and Frequency. The Execution subscale measured days per week during which foot self-care was performed. This part included four questions; each was rated as follows: 1 = 0 days, 2 = 1–2 days, 3 = 3–4 days, 4 = 5–6 days, and 5 = 7 days. The Frequency subscale (summarizing three questions) categorized the frequency of foot self-care behavior. Those were rated as follows: 1 = never, 2 = sometimes, 3 = seldom, 4 = often, and 5 = always. A higher score of the subscale of execution or frequency means a good diabetes foot self-care behavior. The DFSBS had been validated in Taiwan; the Internal consistency of Cronbach’s alpha values was 0.73 [7]. Internal consistency Cronbach’s alpha value of this study was 0.91, which meant acceptable reliability for this scale.

### 2.3. Data Collection and Analysis

The ethics committee approved this study (IRB No. 104009). Patients were recruited in the outpatient clinics of the endocrine department in Taiwan between 1 May 2015 and 30 May 2016. The data were collected after informed consent was obtained. Although the participants had signed the consent form, they still had the right to withdraw from the study at any time. The data anonymization strategy was adopted to protect the participants’ privacy and rights, and the collected data were only used for the purposes of this research.

Descriptive statistics, including frequency, percentage, mean, and standard deviation, were used to understand sample distributions of demographic, disease attributes, the diabetic foot self-care behavior scale (DFSBS), and the diabetic foot ulcer health belief scale (DFUHBS). The Pearson correlation test was used to examine the univariate correlation between diabetic foot ulcer health beliefs and self-care behavior. Multivariable linear regression was used to examine the association between diabetic foot self-care behavior and foot ulcer health beliefs (model I: Diabetic foot self-care behavior total scale; model II: Execution subscale; model III: Frequency subscale) by using significant factors from the Pearson correlation test. Residence (living with family or living alone), gender, and the number of years people had type II Diabetes Mellitus (DM duration), were set as confounding factors in the regression model. The significance level was set at *p* < 0.05 and was two-tailed. The SPSS for Windows software, version 20.0 (SPSS Inc., Chicago, IL, USA), was used for the data analysis. 

## 3. Results

### 3.1. Descriptive Statistics of Study Participants 

The participants were mostly younger than 65 years old (58.2%) and male (54.1%). Their education level was mainly lower than junior high school (54.1%), and most of the participants were unemployed or retired (71.4%). In addition, the majority of participants lived with their families (87.8%) and were married or widowed (93.9%). As for disease attributes, 70.4% of the participants had a diabetes diagnosis for less than ten years, 67.3% had a BMI greater than 24, and 69.4% had HbA1c greater than 7%.

The participants’ average total DFSBS self-care score was 19.19 out of 35 points. The average subscale scores were 10.45 out of 20 on the execution subscale and 8.74 out of 15 points on the behavior frequency subscale (see Table 1).

The participants’ average Health Belief score on the total DFUHBS was 122.61 points out of 175. On the subscale of benefit beliefs, the participants’ average score was 44.19 points out of 60. The subjects’ average score for threat beliefs was 31.74 points out of 45, and the average score on the barrier beliefs subscale was 46.47 points out of 70. A higher score means a stronger health belief (see Table 2).

### 3.2. Disparities of Self-Care (DFSBS), among Demographic and Disease Attribute Groups 

The results showed that the average total DFSBS score of the subjects living with families was significantly higher than those living alone (*p* = 0.012). As for disease attributes, subjects with a duration of diabetes ten years or greater had an average total DFSBS score significantly higher than those with diabetes less than ten years (*p* = 0.029). However, other demographic variables and disease attributes did not show statistically significant differences (see Table 1). Only the residence status has significant differences in the execution subscales (*p* = 0.020). However, with the frequency subscale, the subjects living with families had significantly higher average scores than those living alone (*p* = 0.004). Female subjects had significantly higher average scores than males (*p* = 0.042). Subjects with a duration of diabetes ten years or greater had significantly higher average scores than among those with diabetes duration less than ten years (*p* = 0.036) (see Table 1).

### 3.3. Disparities of Health Beliefs (DFUHBS), among Demographic and Disease Attribute Groups 

There were no significant differences in average scores of the total DFUHBS, or the threat or barrier subscales, among demo-graphic or disease attribute groups. However, the subjects with junior high school or lower educational level showed significantly lower average scores of the benefit beliefs subscale than the subjects with senior high school or higher educational level (*p* = 0.032). For subjects who had diabetes less than ten years, the average score of benefit beliefs was significantly lower than subjects who had diabetes for ten years or more (*p* = 0.049) (see Table 2).

### 3.4. The Correlation between the Diabetic Foot Self-Care Behavior Scale (DFSBS) and the Diabetic Foot-Ulcer Health Belief Scale (DFUHBS)

There was a significantly positive correlation between total DFUHBS and total DFSBS (r = 0.58, *p* < 0.01). Further analysis showed that diabetic foot self-care behavior (total DFSBS) had a significant positive correlation with benefit beliefs (r = 0.59, *p* < 0.01), threat beliefs(r = 0.22, *p* < 0.05), and barrier beliefs(r = 0.51, *p* < 0.01) (see Table 3). The number of days of foot self-care performing behavior within each week (Execution) had a significant positive correlation with the total DFUHBS (r = 0.48, *p* < 0.01), as well as the benefit belief (r = 0.52, *p* < 0.01) and barrier belief subscales (r = 0.44, *p* < 0.01) (see Table 3). The frequency of foot self-care behavior (Frequency) also had a significant positive correlation with the total DFUHBS (r = 0.61, *p* < 0.01), benefit beliefs (r = 0.57, *p* < 0.01), threat beliefs (r = 0.31, *p* < 0.01), and barrier beliefs(r = 0.51, *p* < 0.01) (see Table 3).

### 3.5. Predicting Diabetic Foot Self-Care Behavior (DFSBS) from Demographic, Disease Attributes, and Diabetic Foot Ulcer Health Beliefs (DFUHBS)

After controlling for diabetes duration and threat beliefs covariates, the subjects’ residence status (β = 0.19, *p* < 0.05), benefit beliefs (β = 0.40, *p* < 0.01), and barrier beliefs (β = 0.24, *p* < 0.05) were significantly associated with total DFSBS score, and the entire model explained 42.9% of the variance of DFSBS (see Table 4, Model I). Furthermore, we analyzed the significant predictors of the number of behavior-performing days within each week (Execution subscale). The results indicated that controlling for threat beliefs, barrier beliefs, and benefit beliefs (β = 0.42, *p* < 0.01) was significantly associated with self-care execution, and the entire model explained 31.8% of the variance of the number of performing days within a week (see Table 4, Model II). Finally, controlling for gender, diabetes duration, and threat beliefs covariates, we analyzed the significant predictors of the behavior frequency within each week (Frequency subscale). The results indicated that the subjects’ residence status (β = 0.21, *p* < 0.01), benefit beliefs (β = 0.32, *p* < 0.01), and barrier beliefs (β = 0.27, *p* < 0.01) were significantly associated with self-care frequency, and the entire model explained 46.8% of the variance of the frequency subscale (see Table 4, Model III).

## 4. Discussion

Diabetes foot self-care behavior is an important issue. As a result, several countries have begun developing and validating the tool for diabetes foot self-care behavior assessment under different cultural contexts [9,10]. Based on the execution and frequency of diabetes foot self-care behavior, this study found that patients who live with families had significantly better diabetic foot self-care behaviors. Like our findings, qualitative and quantitative studies found that family and friends can help patients manage diabetes, which points out that social support is essential for promoting diabetes self-care behavior [17,18]. For patients living with family, the overall family function is relatively complete, and they supposedly had higher social support, which might be related to self-care behavior performance. 

Patients with a longer duration of diabetes had better diabetic foot self-care behavior. This was consistent with previous research [19]. In other studies, those with lower body weight showed better diabetic-foot self-care behaviors [20], and those with better overall self-care behavior had lower glycated hemoglobin [21]. However, another study found no significant correlation between the duration of diabetes and self-care behaviors [18]. One possible reason for this finding could be that those who had diabetes for a long time received the care of more integral medical systems, which motivated them to pay better attention to their diabetes and focus on their self-care behavior. That self-care behavior could have helped those subjects to control their body weight and glycated hemoglobin levels.

The results showed that the diabetic foot self-care behavior was significantly and positively correlated with diabetic foot ulcer health beliefs. Each subscale (benefit, threat, and barrier beliefs) was positively correlated with diabetic foot self-care behaviors. Further analysis of the self-care behavior subscale of frequency and the number of performing days within a week showed a positive correlation to the belief, but only the threat belief was inconsistent.

Positive health beliefs about diabetes are expected to improve patient’s compliance with their treatment. A similar result also showed that applying the health belief model to the self-care of diabetic patients had a significant influence on health behavior [12,22]. According to another study on diabetes based on the health belief model, higher threat beliefs correlated with better diabetic self-care behavior [23]. However, the study showed that threat belief was not an influence on health behavior (the number of performing days within a week). Similarly, the research has also demonstrated that threat beliefs did not significantly correlate with diabetic foot self-care behavior [13]. A meta-analysis showed a positive relationship between perceived disease severity and compliance with treatment, but compliance was affected when the patient’s health was impaired [24]. The reason for this might be that the health beliefs in the present study were chiefly concerned with the benefit beliefs and whether they could be related to a reduced incidence or deterioration of foot ulcers. Therefore, similar to the previous study’s results, there was no consistent conclusion regarding threat beliefs.

This study also found that having cohabitants, higher benefit, and barrier beliefs were significant predictive factors of overall diabetic foot self-care behavior. The literature points out that social support is an essential factor for promoting self-care behavior [25], and patients with better overall family function have better self-care behavior [18]. Based on the literature and the present findings, it is clear that there is a positive relationship between health beliefs about self-care for diabetic feet and compliance with treatment [23].

The benefit beliefs influenced the diabetic patients’ behavior of regularly seeking medical care. Benefit beliefs were also an important predictive factor for the number of implementation days within each week, while having cohabitants, benefit beliefs, and barrier beliefs were significant predictive factors for behavior frequency. Stronger benefit beliefs related to health behavior correspond to better treatment and prevention behaviors [12,14]. Patients are more willing to practice regular self-care behavior when they think that self-care would benefit their diseases.

Some studies indicated that those who perceived higher susceptibility, severity, behavior benefits, and lower behavior barriers significantly perform more preventive health behaviors [12,13]. Thus, perceived barrier beliefs had a negative correlation with health behavior. Interestingly, our study showed that patients with higher perceptions of disease threat had higher health behavior barriers and better prevention behaviors. Patients’ perceived barrier beliefs were a significant factor in predicting diabetic foot self-care behavior and behavior frequency. Similar results also showed that patients with higher disease threat beliefs had higher health-behavior barriers and better prevention behaviors [12,25]. This phenomenon might be that the participants were type II diabetic patients who were all seeking medical care regularly. It can be deduced that these patients would have a higher perception of disease threat, and most had suffered from diabetes for more than ten years. Although they had barrier beliefs, their disease-threat perception still was also firmer. Those might contribute to better self-management behavior, and the results show that barrier beliefs can positively predict better diabetic foot self-care behavior.

## 5. Limitations of Study

There are several limitations to this study. First, the study recruited patients with type II diabetes to assess the health beliefs related to diabetic foot ulcers and diabetic foot self-care behaviors at a medical center in Central Taiwan. The study findings may not be generalizable to other geographic regions in Taiwan or patients who seek medical care in different settings. Second, this study used a structured questionnaire to investigate the health beliefs regarding diabetic foot ulcers and foot self-care behaviors, evaluation by self-report. Any recalled bias may have resulted in overestimation or underestimation of the behaviors. Third, due to the small sample size of our study, the findings might have been different if there were more participants and a variety of settings. 

Thus, future studies should recruit more representative random samples that could be collected or various geographic settings. Moreover, patients who have diabetes and are failing to seek regular medical care could be included in future study populations. Lastly, the representativeness and validity of the data could be improved by using more structured assessment tools to collect data; more qualitative and quantitative research could be conducted to increase empirical data.

## 6. Conclusions

We found that living alone, shorter duration of diabetes, male gender, and lower health belief scores predict less adequate diabetic foot self-care behavior. Health care providers should assess these factors when designing individual care plans.

## Figures and Tables

**Table 1:** Disparities of self-care DFSBS results among demographic and disease attribute groups.

Variable	*n*	Total DFSBS Mean ± SD	*p*	Execution Mean ± SD	*p*	Frequency Mean ± SD	*p*
	98	19.19 ± 8.61		10.45 ± 5.62		8.74 ± 3.75	
Age			0.192		0.223		0.243
<65	57	18.23 ± 8.38		9.86 ± 5.47		8.37 ± 3.67	
≥65	41	20.54 ± 8.85		11.27 ± 5.80		9.27 ± 3.83	
Education			0.868		0.969		0.748
≤Junior high school	51	19.33 ± 8.30		10.47 ± 5.37		8.86 ± 3.61	
≥Senior high school	47	19.04 ± 9.02		10.43 ± 5.94		8.62 ± 3.93	
Occupation			0.060		0.064		0.124
Unemployed/retired	70	20.23 ± 8.27		11.11 ± 5.52		9.11 ± 3.54	
Employed	28	16.61 ± 9.05		8.79 ± 5.6		7.82 ± 4.15	
Residence			**0.012**		**0.02**		**0.004**
Living alone	12	13.42 ± 6.30		7.58 ± 3.90		5.83 ± 2.86	
Live with families	86	20.00 ± 8.61		10.84 ± 5.72		9.15 ± 3.69	
Marital status			0.733		0.798		0.722
Single	6	14.17 ± 11.63		10.69 ± 6.04		9.00 ± 4.47	
Married or widowed	92	19.52 ± 8.36		10.36 ± 5.50		8.65 ± 3.48	
Gender			0.150		0.394		**0.042**
Male	53	18.04 ± 8.49		10.00 ± 5.57		8.04 ± 3.74	
Female	45	20.55 ± 8.65		10.98 ± 5.69		9.58 ± 3.62	
DM duration			**0.029**		0.053		**0.036**
<10 years	69	17.97 ± 8.39		9.74 ± 5.50		8.23 ± 3.60	
≥10 years	29	22.10 ± 8.57		12.14 ± 5.65		9.97 ± 3.88	
BMI			0.945		0.898		0.725
<24	32	19.28 ± 9.33		10.34 ± 5.80		8.94 ± 3.98	
≥24	66	19.15 ± 8.32		10.50 ± 5.58		8.65 ± 3.66	
HbA1c			0.727		0.773		0.713
<7%	30	18.73 ± 7.87		10.20 ± 5.29		8.53 ± 3.47	
≥7%	68	19.40 ± 8.97		10.56 ± 5.79		8.84 ± 3.88	

DFSBS: Diabetic foot self-care behavior scale, Execution: Number of days performing foot self-care within a week, Frequency: Frequency of behaviors performing foot self-care within a week, DM duration: the number of years since the first diagnosis of type II Diabetes Mellitus, BMI: body mass index, HbA1c: glycated hemoglobin.

**Table 2:** Disparities of Health Beliefs (DFUHBS), among demographic and disease attribute groups.

Variable	*n*	Total DFHBS Mean ± SD	*p*	Benefit Mean ± SD	*p*	Threat Mean ± SD	*p*	Barrier Mean ± SD	*p*
Age			0.501		0.918		0.080		0.911
<65	57	124.02 ± 19.04		44.28 ± 8.67		33.18 ± 7.74		46.56 ± 9.13	
≥65	41	120.66 ± 27.34		44.07 ± 11.16		29.76 ± 10.43		46.83 ± 13.10	
Education			0.162		**0.032**		0.329		0.835
≤Junior high school	51	119.51 ± 22.02		42.18 ± 9.30		30.88 ± 9.78		46.45 ± 10.83	
≥Senior high school	47	125.98 ± 23.41		46.38 ± 9.81		32.68 ± 8.24		46.91 ± 11.11	
Occupation			0.960		0.418		0.748		0.748
Unemployed or retired	70	122.69 ± 21.12		44.70 ± 9.25		31.56 ± 8.85		46.43 ± 11.01	
Employed	28	122.43 ± 26.99		42.93 ± 10.92		32.21 ± 9.77		47.29 ± 10.82	
Residence			0.402		0.339		0.841		0.464
Living alone	12	117.42 ± 29.52		41.67 ± 13.57		31.25 ± 11.58		44.50 ± 10.87	
Live with families	86	123.34 ± 21.83		44.55 ± 9.12		31.81 ± 8.75		46.98 ± 10.94	
Marital status			0.842		0.888		0.212		0.624
Single	6	123.38 ± 27.63		43.96 ± 11.95		33.65 ± 8.91		45.77 ± 11.72	
Married or widowed	92	122.33 ± 21.02		44.28 ± 8.89		31.06 ± 9.09		47.00 ± 10.67	
Gender			0.582		0.946		0.431		0.662
Male	53	121.43 ± 25.47		44.13 ± 10.42		31.08 ± 9.76		46.23 ± 11.37	
Female	45	124.00 ± 19.41		44.27 ± 8.96		32.53 ± 8.23		47.20 ± 10.44	
DM duration			0.077		**0.049**		0.562		0.166
<10 years	69	119.97 ± 22.89		42.94 ± 10.28		31.35 ± 8.09		45.68 ± 10.56	
≥10 years	29	128.90 ± 21.73		47.17 ± 7.63		32.69 ± 11.18			
BMI			0.869		0.595		0.407	49.03 ± 11.54	0.576
<24	32	122.06 ± 25.00		43.44 ± 10.34		32.84 ± 9.52		45.78 ± 11.82	
≥24	66	122.88 ± 21.87		44.56 ± 9.48		31.21 ± 8.87		47.11 ± 10.50	
HbA1c			0.141		0.361		0.226		0.213
<7%	30	117.50 ± 22.16		42.83 ± 9.91		30.07 ± 7.75		44.60 ± 11.36	
≥7%	68	124.87 ± 22.89		44.79 ± 9.67		32.49 ± 9.56		47.59 ± 10.66	

DFHBS: Diabetic foot ulcer health belief scale, DM duration: the number of years people with type II Diabetes Mellitus, BMI: body mass index, HbA1c: glycated hemoglobin.

**Table 3:** Pearson correlation (r) between diabetic foot ulcer health beliefs and self-care behavior.

Variables	Total DFSBS ^a^	Execution ^c^	Frequency ^d^
Total DFUHBS ^b^	0.58 **	0.48 **	0.61 **
Benefit belief	0.59 **	0.52 **	0.57 **
Threat belief	0.22 *	0.13	0.31 **
Barrier belief	0.51 **	0.44 **	0.51 **

* *p* < 0.05, ** *p* < 0.01, ^a^ DFSBS: Diabetic foot self-care behavior scale. ^b^ DFHBS: Diabetic foot ulcer health belief scale. ^c^ Execution: Number of days performing foot self-care within a week. ^d^ Frequency: Frequency of performing foot self-care within a week.

**Table 4:** Multivariable linear regression models predicting diabetic foot self-care behavior.

	95% CI of B						
**Variables**	**B**	***β***	**SE**	**Lower**	**Upper**	**R^2^**	***p*-Value**
Residence	4.987	0.19	2.070	0.88	9.10		**0.018**
DM duration	1.843	0.10	1.510	−1.16	4.84		0.225
Benefit	0.354	0.40	0.090	0.17	0.54		**0.000**
Threat	0.012	0.01	0.083	−0.15	0.18		0.882
Barrier	0.192	0.24	0.077	0.04	0.35		**0.015**
Model I (dependent: Total DFSBS)						0.429	
Residence	2.346	0.14	1.468	−0.57	5.26		0.113
Benefit	0.244	0.42	0.067	0.11	0.38		0.000
Threat	−0.043	−0.07	0.059	−0.16	0.07		0.462
Barrier	0.097	0.19	0.055	−0.01	0.21		0.080
Model II (dependent: Execution subscale)						0.318	
Gender	1.126	0.15	0.583	−0.03	2.28		0.056
Residence	2.396	0.21	0.886	0.64	4.16		**0.008**
DM duration	0.788	0.10	0.638	−0.48	2.06		0.220
Benefit	0.124	0.32	0.040	0.04	0.20		**0.003**
Threat	0.048	0.12	0.635	−0.02	0.12		0.170
Barrier	0.093	0.27	0.033	0.03	0.16		**0.006**
Model III (dependent: Frequency subscale)						0.468	

Dependent variable: Model I: Diabetic foot self-care behavior total scale, Model II: Diabetic foot self-care behavior-Execution subscale, Model III: Diabetic foot self-care behavior-Frequency subscale, Residence: 1 = living with family, 0 = living alone, DM duration: 1 = ten years or more, 0 = less than ten years, Gender: 1 = female, 0 = male.

## Data Availability

The data presented in this study are available on request from the corresponding author. The data are not publicly available due to privacy.
